# Retraction Note: Fetal echocardiographic parameters in pregnancies complicated by diabetes: a case control study

**DOI:** 10.1186/s12884-026-08812-z

**Published:** 2026-02-14

**Authors:** Amal Darwish, Maged Abdel-Raouf, Rasha Kamel, Emad Salah, Mai Salah, Ahmed Okasha

**Affiliations:** 1 Department of Obstetrics and Gynecology, Faculty of Medicine, Kasr El Aini Hospital, Cairo University, Cairo, Egypt; 2 Maternal Fetal Medicine Unit, Department of Obstetrics and Gynecology, Kasr El Aini Hospital, Cairo University, Cairo, Egypt; 3 Department of Obstetrics and Gynecology, Embaba General Hospital, Egyptian Ministry of Health and Population, Giza, Egypt; 4 Department of Reproductive Health and Family Planning, Medical Research Institute, National Research Centre, Cairo, Egypt

**Retraction Note: BMC Pregnancy and Childbirth 22, 650 (2022)**

**https://doi.org/10.1186/s12884-022-04969-5**

RETRACTION NOTE.

The Editor has retracted this article. Following publication, concerns were raised about 30 duplications in the authors’ dataset. The authors noted that they had published the wrong dataset and provided a corrected file However, the new dataset contained additional issues including the absence of participants lost to follow up, which were described in the article, differences in rounding of values, and the presence of the same means as in the original dataset without clear explanation as to which data were original and how the error occurred. The Editor has therefore lost confidence in the integrity of the data reported in the article. Ahmed Okasha has stated on behalf of the authors that they agree to this retraction.

